# Induction of oil accumulation by heat stress is metabolically distinct from N stress in the green microalgae *Coccomyxa subellipsoidea* C169

**DOI:** 10.1371/journal.pone.0204505

**Published:** 2018-09-27

**Authors:** James W. Allen, Rahul Tevatia, Yaşar Demirel, Concetta C. DiRusso, Paul N. Black

**Affiliations:** 1 Department of Biochemistry, Beadle Center, University of Nebraska-Lincoln, University of Nebraska-Lincoln, Lincoln, NE, United States of America; 2 Department of Chemical and Biomolecular Engineering, Othmer Hall, University of Nebraska-Lincoln, Lincoln, NE, United States of America; Universidad Nacional Autonoma de Mexico Centro de Nanociencias y Nanotecnologia, MEXICO

## Abstract

Algae are often promoted as feedstock organisms to produce a sustainable petroleum fossil fuel alternative. However, to induce lipid accumulation most often requires a severe stress that is difficult to induce in large batch cultures. The objective of this study is to analyze and mathematically model heat stress on growth, chlorophyll content, triacylglyceride, and starch synthesis in algae. We initially screened 30 algal species for the most pronounced induction of lipid droplets from heat stress using confocal microscopy and mass spectroscopy techniques. One species, *Coccomyxa subellipsoidea* C169, was selected and subjected to further biochemical analyses using a jacketed bioreactor amended with 1% CO_2_ at 25°C, 30°C, 32°C, 33°C, 34°C, 35°C, and 36°C. Lipid and starch accumulation was less extreme than N stress. Growth was reduced above 25°C, but heat stress induced lipid droplet synthesis was negatively correlated with growth only past a demonstrated threshold temperature above 32°C. The optimal temperature for lipid accumulation was 35°C, which led to 6% of dry weight triglyceride content and a 72% reduction from optimal growth after 5 days. Fatty acid influx rates into triglycerides and ^15^N labeling of amino acids and proteins indicate that heat stress is mechanistically distinct from N stress. Thus, this study lends support to a novel hypothesis that lipid droplet triglycerides result from a redistribution of carbon flux as fatty acids to neutral storage lipids over membrane or other lipids.

## Introduction

Replacing petroleum fuels with algal biofuels leads to a beneficial cycle, offsetting anthropogenic CO_2_ released through combustion by capturing atmospheric or industrial CO_2_ as algal biomass in real time [1]. This technology shifts the net outcome of fuel production away from petroleum refining pollution and towards the consumption of nutrients and the sequestration of CO_2_ into stable algal biopolymers. Coupling algae growth with municipal wastewater flows reduces nitrate and phosphate pollution, preventing the growth of detrimental photosynthetic organisms such as toxin-producing cyanobacteria [2, 3]. Algal protein and cell wall material can also be added to animal feed, directly administered to agricultural land as a biochemical fertilizer, or used as industrial chemical feed-stocks in the production of high value commodities [4–7].

The implementation of algal biofuels is currently hampered by commonly suboptimal growth rates, energy costs (*e*.*g*. harvesting and dewatering), and very low oil yields typical of wild type algae in the absence of abiotic stress [8, 9]. Among these issues, determining and reversing the biochemical constraints of oil synthesis in algae, either through biotechnology or mass culture methodology, may be a critical key to the bioeconomy. Several stress conditions result in the intracellular synthesis and accumulation of triacylglyceride (TAG) filled lipid droplets (LDs) in algae [10–12], and can lead to relative dry biomass oil percentages greater than agricultural seeds [13]. Nitrogen (N) starvation is especially effective in producing very high oil content algae, and is the most widely used method for inducing TAG accumulation in the laboratory. However, this method is untenable with large scale culturing since it requires a two-stage system and an intermediate dewatering step to remove sources of N, effectively doubling collection costs [14]. The determination and genetic manipulation of biochemical factors leading to oil accumulation could result in strains of algae that constitutively accumulate oil. Alternatively, methods can be developed to remove N without dewatering, or to use an equivalent stress unrelated to the chemical makeup of the growth media.

The net effect of N limitation on algae is the replacement of new cell creation with oil and/or starch synthesis. Chemostatic continuous culture studies using N as a limiting resource have shown a progressive influence on growth rate and oil accumulation in algae, with one gradually increasing as the other declines [10]. While this is an apparently intrinsic quality of algae, there is some evidence that storage compound accumulation can also be induced through the direct genetic manipulation of biochemical pathways. For example, Haimovich-Dayan *et al*. [15] recently demonstrated that a knock-down of chloroplastic pyruvate-orthophosphate dikinase (PPDK) in *Phaeodactylum tricornutum*, catalyzing the conversion of pyruvate to phosphoenolpyruvate (PEP), increases accumulation of both fatty acids and carbohydrates with little effect on growth. Similarly, the knockdown of a Zn(II)2Cys6 transcriptional factor identified in *Nannochloropsis gaditana* results in lipid accumulation while maintaining normal growth [16]. These studies indicate that energy storage can co-occur with the production of new cells under metabolic regulation.

Further discovery of conditions redirecting metabolic flux into an oleaginous state would greatly benefit from highlighting regulatory pathways specific to its induction. Global transcriptional and proteomic comparisons across types of stress leading to TAG accumulation could lead to trait specific pathways much like comparative analyses of disparate photosynthetic organisms led to the GreenCut proteins, which isolated photosynthesis-specific processes [17]. Currently there is a small body of literature demonstrating that heat stress can lead to lipid accumulation. A doubling of total lipids was shown in *Nannochloropsis oculata* when increasing temperature from 20°C to 25°C, and a greater than fivefold increase in TAG content in *Ettlia oleoabundans* during exposure to 35°C [18, 19]. Heat stress could greatly inform mechanisms of lipid accumulation caused by N stress, especially if it is found to be based on fundamentally different mechanisms. Since intense solar irradiance needed to support large scale algal agriculture is necessarily coincident with hotter ambient temperatures, heat stress may also be a more realistic effector than N stress for increasing oil yields through a two stage industrial process. Although heat stress may provide an alternative condition for either comparative analyses or an industrially relevant finishing treatment, little is currently known about the phenomenon or its generality among algal species.

In the current study, a phenotypic survey of 42 alga comprising 30 species was conducted analyzing the production of LD’s resulting from mild heat stress induced at 32°C in shake flasks, using confocal microscopy and mass spectroscopy. The initial screen resulted in six algae which produced LDs with varying degrees of lipid accumulation. Repeated testing in a temperature controlled, high CO_2_ bioreactor system resulted in TAG accumulation by only one of the six initial positives, a cold-tolerant model biofuel species *Coccomyxa subellipsoidea* C169 [20]. Using this species, the relationships between heat stress, growth, chlorophyll content, TAG and starch synthesis were further analyzed by a series of time courses varying temperature only. Results were analyzed in comparison to N starvation and temperature specific data mathematically modeled. Labeling studies demonstrated that heat stress was unrelated to N assimilation into amino acids or proteins, and the rates of fatty acid (FA) input into TAG pools showed that oil accumulated due to flux redistribution rather than increased FA synthesis. Heat stress accumulated 40% of the TAG compared to N stress on a per cell basis and appears to be mechanistically distinct, making it a useful condition for comparative analyses.

## Materials and methods

### Organisms, culturing conditions, and screening tests

*Coccomyxa subellipsoidea* C169 was obtained from the laboratory of James Van Etten (Department of Plant Pathology, University of Nebraska-Lincoln), originally obtained from the Microbial Culture Collection, National Institute for Environmental Studies, Japan (NIES 2166). Origins and strain designations of other algae tested are referenced in Table 1. For the initial screen, algae were transferred from plate cultures to 30mL of modified Bold’s Basal Medium (BBM) containing a three times normal amount of sodium nitrate (BBM-3N), and grown at 25°C in an Innova 43 lighted shaking incubator (New Brunswick Scientific) set to 125 RPM. The temperature was shifted to 32°C after 3 days of initial growth and maintained for one week. LD accumulation of was visualized after treatment using Nile Red staining. Confocal laser scanning microscopy was performed with a Nikon A1 CLSM mounted on a Nikon 90i compound microscope and controlled by NIS-Elements (5.02.00) with a 100x objective. All data was collected sequentially to prevent signal cross-talk. The transmitted light data was collected with the 640.9 nm laser. Excitation and emission wavelengths for Nile Red visualization were 561.6 nm and 570–620 nm, pseudo-colored red in images. Where applicable, the chlorophyll fluorescence was analyzed using 640.9 nm and 663–738 nm for excitation and emission, respectively, and pseudo-colored green.

**Table 1:** Species list of algae used for phylogeny screening of lipid accumulation in mild heat stress.

Class Trebouxiophyceae		Class Chlorophyceae	
***C*. *emersonii***	CCAP 211/8a	***A*. *superbus***	UTEX 88
***C*. *emersonii***	UTEX 2337	***Ch*. *reinhardtii***	CRC CC-125
***C*. *kessleri***	UTEX 1808	***Ch*. *reinhardtii***	CRC CC-4348
***C*. *minutissima***	UTEX 2341	***S*. *basiliensis***	UTEX 79
***C*. *mirabilis***	SAG 211-11r	***S*. *breviaculeatus***	UTEX 2443
***C*. *parva***	UTEX B1805	***S*. *naegelii***	UTEX 74
***C*. *protothecoides***	UTEX 29	***S*. *abundans***	UTEX 1358
***C*. *protothecoides***	UTEX B264	***S*. *acutus***	UTEX B72
***C*. *saccharophila***	SAG 3.8	***S*. *bijugatus***	UTEX B413
***C*. *sorokiniana***	UTEX 1230	***S*. *dimorphus***	UTEX 1237
***C*. *sp*.**	UTEX BSNO60	***S*. *dimorphus***	UTEX 417
***C*. *sp*.**	UTEX BSNO69	***S*. *obliquus***	UTEX 1450
***C*. *viscosa***	SAG 2338	***S*. *obliquus***	UTEX 393
***C*. *vulgaris***	UTEX 265	***S*. *obliquus***	UTEX B2630
***C*. *vulgaris***	UTEX 395	***S*. *parisiensis***	UTEX 1584
***C*. *aciculais***	UTEX LB1381	***S*. *parisiensis***	UTEX 1585
***Co*. *subellipsoidea***	NIES 2166	***S*. *parisiensis***	UTEX 1586
***D*. *splendida***	UTEX LB1381	***S*. *sp*.**	UTEX 2193
***P*. *muralis***	UTEX 2254	***St*. *pascheri***	UTEX LB1571
***T*. *sp*.**	UTEX 2453	**Class Xanthophyceae**	
***W*. *reniformis***	UTEX 2912	***B*. *filformis***	CCALA 224
		***Tr*. *aequale***	NCMA 2166

Genus abbreviations: A., Asterococcus; B., Bumilleriopsis; C., Chlorella; Ch., Chlamydomonas; Co., Coccomyxa; D. Dictyochloropsis; P., Palmellopsis; S., Scenedesmus; St., Stigeoclonium; T., Tetrachlorella; Tr., Tribonema; W., Watanabea.

Culture collections: CCALA, Culture Collection of Autotrophic Organisms, Czech Republic; CRC, Chlamydomonas Resource Center, USA; NCMA, National Center for Marine Algae and microbiota, USA; NIES, National Institute for Environmental Studies, Japan; SAG, Culture Collection of Algae at Göttingen University, Germany; UTEX, Culture Collection of Algae at the University of Texas Austin, USA.

Secondary screening analyses were conducted in technical triplicate in 6 L batch cultures in axenic conditions using jacketed cylindrical bioreactors with overhead drive motors and paddle-type stirring at a rate of 75 RPM (Bellco Glass, Inc.). Flasks, media and bioreactors were sterilized by autoclaving. High-resolution correlated gas flow meters (Cole-Parmer) were used to control aeration of compressed air (2 L min^-1^) supplemented with CO_2_ supplied from a tank at a concentration of 1% v/v. Growth was photo-autotrophic with 200 μmols photons m^-2^s^-1^ provided by conventional 200 Watt equivalent CFM bulbs. Inoculum for each bioreactor was grown for 5 days in six 35 mL cultures contained in 250 mL flasks in a lighted shaking incubator as already described. A 6 L bioreactor was inoculated to an initial OD_550_ of 0.03 ± 0.01 and grown at room temperature for 5 days. Pre-temperature controlled growth was monitored for consistency. The OD_550_ was adjusted downward if necessary by the replacement of media with sterile BBM and temperature control was initiated by the attachment of a Neslab RTE7 circulating water bath (Thermo Scientific) set to 35°C. This was maintained for five days. Four 50mL samples were taken daily, centrifuged, and pellets stored at -80°C until lyophilized, weighed and analyzed.

#### Culturing for biochemical analyses

Further biochemical analyses were done using the bioreactor vessels, conditions, and sample collections already described, but varying temperature to 25°C, 30°C, 32°C, 33°C, 34°C, 35°C or 36°C. Temperature studies were conducted in biological triplicate. Additional biological triplicate cultures were grown for comparison studies between heat and N starvation at 25°C. N starved cultures were started at an OD_550_ of 1 ± 0.15 and fluorescent light was doubled to compensate for the increased algal density. Nitrogen starvation was achieved by removing and centrifuging the cultures, washing the pellets with sterile BBM media modified to exclude a nitrogen source (N-BBM), and re-suspending the algae into 6 L of N-BBM.

Amino acid and protein analyses were done in biological triplicate where the initial growth in 3 L batches of BBM containing the normal amount of unlabeled sodium nitrate (3 mM). Cultures were collected into sterile bottles at an OD_550_ of 1, diluted two fold using N-BBM 򠁮d transferred to two sterilized 6 L bioreactors. Stable isotopic labeling was added as a probe for relative *de novo* synthesis of amino acids and their incorporation into proteins using a final concentration of 1 mM of ^15^N-ammonium chloride when temperature control was initiated. One bioreactor batch culture was kept at room temperature and the other heated (35°C) for each biological replicate. Bioreactor conditions were as previously described. 200 mL volumes of each culture were collected after 24hrs, centrifuged at 4°C, frozen in liquid N_2_ and lyophilized.

#### Studies on incremental temperature increases

Cultures were grown in biological quadruplet to an OD_550_ of 0.5 in 3N-BBM at 25°C. An initial 1 day further at 25°C was used as a baseline followed by 1 day intervals at 30°C, 32°C, 34°C, and 36°C. At the end of each 24hr growth phase, the cultures were adjusted back to an OD_550_ of 0.5 by syphoning off and replacing media. This controlled for possible changes due to reduced light transmission from cultures growing denser over time.

#### Analysis of starch and chlorophyll content

Cellular starch contents were analyzed using the procedure of Brányiková *et al*. [21] with some modifications. A more stringent cell disruption step was required and the pigments removed prior to starch extraction as they were found to interfere with the spectrophotometric measurements. Lyophilized algal samples were weighed and bead milled in 1.5 mL microcentrifuge tubes with 300 mg of 0.7 mm zirconia beads (Analspec) using a Qiagen TissueLyser LT at 50 Hz for 5 min and vortexed 30 sec in 1 mL of cold 100% ethanol. The ethanol was centrifuged and removed, and the process was repeated until color could no longer be visually detected after centrifugation. The samples were re-suspended in water, transferred to glass vials, and lyophilized to remove the remaining ethanol. Starch was extracted and hydrolyzed using three successive 3.3 mL additions of 30% aqueous perchloric acid followed by 15min of vortexing at room temperature, centrifugation at 3000RPM for 5 min, and removal of the supernatant to a collection vial chilled by an ice bath. For starch-anthrone derivitization, 0.5 mL of collected extract was added to 2.5 mL anthrone solution (2 g of anthrone per liter of 75% (v/v) H_2_SO_4_) and quickly vortexed to mix. Samples were boiled for 8 min and placed on ice to cool to room temperature. The extracts were placed in a covered boiling water bath for 10 min and cooled on ice to room temperature. Absorbance of the samples was read at 625 nm on a UV spectrophotometer, using anthrone solution with a final perchloric acid concentration of 6% as the blank. Starch concentrations were calculated using concentration curves generated from starch powder (Sigma) just prior to each analysis. Chlorophyll concentration analyses were as previously reported [22].

#### Lipid extraction and TAG isolation

Total lipid extractions were performed on freeze dried, pre-weighed algae using a modified version of the Bligh & Dyer [23] method as described in [24]. TAGs were isolated for GC-MS analysis by first re-suspending the total lipid extract in 1 mL of methylene chloride. This was added to a pre-made 300 mg silica column followed by 3 mL of methylene chloride. The eluate containing isolated neutral lipids predominated by TAG was dried as before and the fatty acids converted to corresponding methyl esters for quantitative analysis of percent of dry weight TAG content. The internal standard added was 1,2,3-triheptadecanoyl-sn-glycerol (Nu-Check Prep) at the onset of bead milling and at a concentration of 2μg mg^-1^ DW algae. In general, 5–10 mg of DW algae was extracted per sample.

Methyl esters of saponified TAGs were synthesized for GC-MS analysis using a modified method of Morrison and Smith [25]. Glass vials with PTFE lined caps were used with a heat block set at 100°C for the reaction. Dried extracts of TAG isolated by silica column chromatography were re-suspended in 1 mL of 0.5 M sodium hydroxide solution in methanol, the headspace replaced with argon, and heated for 5min. They were left for 1 min to cool to the touch and 1 mL of 14% boron trifluoride in methanol (Sigma Aldrich) added. The argon headspace was replaced and vials sealed before incubating again at 100°C for 5 min. FAMEs were isolated by partitioning with 2 mL of hexanes and 2 mL of saturated aqueous sodium chloride solution. The samples were vortexed briefly and centrifuged. The upper hexane layer was removed to a fresh vial and dried under N2 gas. The dried FAMEs were finally re-suspended in 300 μL of hexanes, transferred to GC vials and analyzed using an Agilent 7890A/5975C gas chromatograph and triple axis mass spectrometer with an Agilent CP7421 SelectFAME column (200 m x 271 μm x 0.25 μm). A temperature gradient was used to obtain separation. The initial 130°C was held for 10min and increased by 10°C min^-1^ to 160°C. This was held for 7 min, increased by 10°C min^-1^ to 190°C, held 7 min at that temperature, increased again by 10°C min^-1^ to 220°C, held 22min at that temperature, increased again by 10°C min^-1^ to 250°C and finally held there for 17 min. The inlet temperature was 250˚C and inlet pressure set to a constant 62.3 psi. Peaks were identified using a 37 FAME standard (Supelco) and the NIST compound library database. Quantification was done by comparison with the peak area of heptadecenoic acid methyl ester derived from the TAG internal standard added prior to cell disruption. It was confirmed by GC-MS analysis *C*. *subellipsoidea* does not synthesize the 17:1Δ10 fatty acid used for an internal standard.

Molecules per L of growth medium was calculated by first converting analyte peak areas to mols per L by comparison with peak areas of a known quantity of internal standard (TG-17:0) and using the known sample volume. The final number for each fatty acid analyte was obtained by multiplication by Avogadro’s number (6.022 x 10^23^ mol^-1^), and was plotted with respect to growth time (days). Molecules per L of growth medium per day for each fatty acid analyte was derived using the slopes of linear regressions taken from these plots.

#### Stable isotopic labeling studies with ^15^N

Dried samples (30 mg) were transferred to micro-centrifuge tubes with 1 g of 0.7 mm diameter zirconia beads, and bead milled cold for 5 min. Free amino acids were extracted in 1.5 mL of (70:30 v/v) methanol/ 0.01 N HCl, and samples partitioned with 1 mL of chloroform followed by 5 min of centrifugation at 14,000 rpm. Aqueous phases were applied directly to gravity columns containing DOWEX 50X8. These were washed twice with 1 mL of deionized water and amino acids eluted with 1 mL of 3 N ammonium hydroxide solution [26]. The ammonia was removed at room temperature under a stream of N_2_ gas and the samples lyophilized overnight. Derivatization was done with 50 μL of pyridine and 50 μL of N-(tert-butyldimethylsilyl)-N-methyl-trifluoroacetamide (MTBSTFA) heated at 60°C for 1 hr. The samples were evaporated to dryness under N2 gas and dissolved in 100 μL of hexanes before analyzing by GC-MS. The inlet was set at 250°C and oven temperature ramped at 1 ml min^−1^ constant flow from 110°C held for 2 min to 260°C at 10°C min^−1^ held for 5 min. The MS was set to scan from 150–600 m/z at a frequency of 2 Hz.

Total proteins were extracted after bead milling using 0.75 mL of PED buffer (100 mM Tris HCl, 1 mM EDTA, 40% Glycerol, 2% SDS) at room temperature followed by centrifugation. Aqueous phases were split and added to 1.6mL of cold acetone, then kept at -20°C overnight to precipitate the proteins. Protein pellets were obtained by centrifugation for 10 min at 15000 xg and removal of the supernatants. The total protein pellets were hydrolyzed by sealing them in 0.5 mL of 6 N HCl under argon gas in glass ampules and heating to 110°C on a heat block for 24 hr. The samples were neutralized with a 12 M sodium hydroxide solution before transferring to cation exchange column as already described.

### Model development

The logistic approach (first-order growth rate differential equation) can be expressed as [27, 28]:

$$\frac{dX(t)}{dt}=\mu_{max}\;\left(1-\frac{X(t)}{X_{max}}\right)\;X(t)$$ (1)

The initial conditions *X*(*t* = 0) = *X*_0_ and *X*(*t* = *t*) = *X*(*t*) provided the analytical solution to Eq (2):

$$X(t)=\frac{{X_{0}X}_{max}e^{\mu_{max}t}}{X_{max}-X_{0}(1-e^{\mu_{max}t})}$$ (2)

where *X*_0_ is initial biomass (mg/L), *X*_*max*_ is maximum biomass, and *μ*_*max*_ is maximum specific growth rate (day^-1^).

To understand the effect of temperature on the growth of *C*. *subellipsoidea* C169, the following assumptions were taken in account: (i) microalgae had a balanced growth i.e. the total amount of cellular compounds is constant, (ii) only one enzyme reaction is rate limiting, (iii) the total amount of rate controlling enzymes per cell is constant, (iv) the reaction rate of the rate limiting enzyme is zero order, and (v) the enzyme reaction shows an Arrhenius type of temperature dependency [28]. These assumptions result in the following expression:

$$\mu_{max}=\mu_{o}\left[\frac{e^{-E_{a}/RT}}{1+ke^{-E_{a}^{*}/RT}}\right]$$ (3)

where *μ*_*o*_ is optimal specific growth rate (h^-1^), *E*_*a*_ is the overall activation energy required by enzymes to support a metabolism in a microalgal cell (J.mol^-1^), $E_{a}^{*}$ is the activation energy required for enzyme denaturation (J.mol^-1^), *R* is the gas constant (J.K^-1^.mol^-1^) and *T* is temperature (°K).

### Product formation kinetics

An unstructured typical kinetic model used widely is the Leudeking-Piret approach, which can model both the growth and non-growth-associated phenomena for product (P) formation of TAG, starch, and chlorophyll [27–29]:

$$\frac{dP(t)}{dt}=\alpha \frac{dX(t)}{dt}+\beta X(t)$$ (4)

Eq (4) shows that product formation rate $\frac{dP(t)}{dt}$ depends linearly on the growth rate and the cell concentration. In this equation, the product formation rate can be contributed by (i) growth related (*α*, mg-product per mg-biomass) coefficient, and (ii) non-growth related (*β*, mg-product per mg-biomass per day) coefficient. Our experimental data indicate that microalgae neutral lipid, starch and chlorophyll production is a result of both growth as well as non-growth–associated phenomena. The analytical solution of Eq (4) for *α* ≠ *β* will be:

$$P(t)=P_{o}+\alpha \;\left(X_{max}-X_{o}+\frac{X_{max}(X_{o}-X_{max})}{{X_{max}+X}_{o}(e^{\mu_{max}t}-1)}\right)+\beta \;\left(\frac{X_{max}(\log[{X_{max}+X}_{o}(e^{\mu_{max}t}-1)]-\log\left[X_{max}\right])}{\mu_{max}}\right)$$ (5)

## Results and discussion

### Phylogenetic analysis of lipid droplet accumulation in microalgae

Evaluating the effects of temperature on lipid desaturation led to the chance discovery that the Arctic alga *C*. *subellipsoidea* C169 accumulates TAG when grown in shake flasks at a mild heat stress of 32°C. Only one other alga strongly exhibiting this trait has been reported [18], leading to the hypothesis that it is more common than currently recognized. The ability to induce TAG accumulation in algae using mild heat stress has implications for the development of algal biofuels, thus a study was undertaken to determine if the phenomenon is phylogenetically widespread, akin to nutrient starvation induction of lipid accumulation, and to find algae suitable for both more detailed biochemical characterization and possible downstream use as a biofuel production strain.

**Fig 1:**
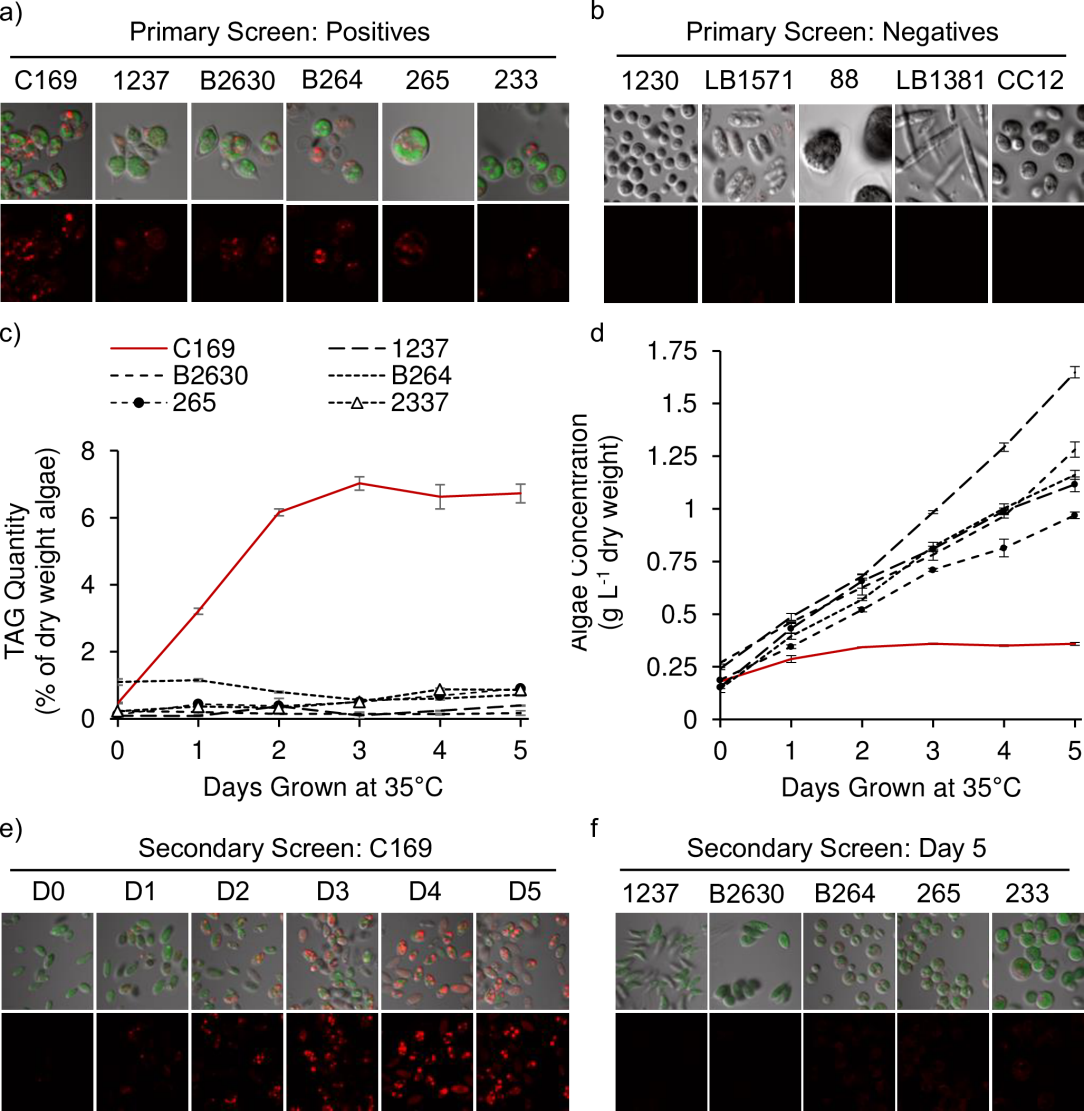
Phylogeny screening of lipid accumulation in algae exposed to mild heat stress. A primary screen of 42 algae grown at ambient CO_2_ for 1 week at 32°C (a-b) and secondary screen in 1% CO_2_ amended bioreactors at 35°C (c-f). (a) Imbedded chlorophyll auto fluorescence (green) and Nile Red fluorescence of lipid droplets (red) in brightfield images (top) and isolated Nile Red fluorescence (bottom) of the six algae positive for lipid droplets in the first screen: *Coccomyxa subellipsoidea* (C169), *Scenedesmus dimorphus* (1237), *Scenedesmus obliquus* (B2630), *Chlorella protothecoides* (B264), *Chlorella vulgaris* (265), and *Chlorella emersonii* (2337). (b) Light and Nile Red fluorescence (top) and isolated Nile Red fluorescence images (bottom) of some negative algae: *Chlorella sorokiniana* (1230), *Stigeoclonium pascheri* (LB1571), *Asterococcus superbus* (88), *Closteriopsis acicularis* (LB1381), and *Chlamydomonas reinhardtii* (CC125). (c) Triglyceride (TAG) content from GC-MS analysis and (d) growth of positive algae for 5 days in high CO_2_ and heat stress. (e) Confocal microscopy of C169 during the secondary screen (days 0–5) and (f) other algae analyzed after 5 days of heat exposure. Error is ± standard deviations (n = 3).

A primary screen was conducted with 42 algae comprising 30 species and ecotypes from our collection of potential biofuel production species using the conditions of initial discovery. Species tested were mostly *Chlorella* and *Scenedesmus* isolates (Table 1). Lipid accumulation as LDs was qualitatively analyzed using confocal microscopy for flask grown cultures after 7 days at 32°C by staining with the lipophilic dye Nile Red. Aside from *C*. *subellipsoidea*, five other possible lipid accumulators were identified based on LD fluorescence: *Scenedesmus dimorphous* (UTEX 1237), *Scenedesmus obliquus* (UTEX B2630), *Chlorella protothecoides* (UTEX 264), *Chlorella vulgaris* (UTEX 265), and *Chlorella emersonii* (SAG 2337) (Fig 1A). LD fluorescence was not otherwise detected (Fig 1B). Three other examples from class Chlorophyceae, *Stigeoclonium pascheri*, *Asterococcus superbus* and *Chlamydomonas reinhardtii*, were not stresses in mild heat and did not accumulate LDs. Two other algae from class Trebouxiophyceae, *Watanabea reniformis* and *Closteriopsis acicularis* also did not produce LDs at 32°C.

Algae identified from the primary screen were further tested in conditions later used for detailed biochemical analyses more closely approximating large scale production photo-bioreactors. A secondary screen was done over 5 days and 1% CO_2_ in a jacketed bioreactor system at 35°C (Fig 1C–1F), a temperature previously shown to induce lipid accumulation in algae [18]. Lipid accumulation and growth were tracked daily by GC-MS analysis of FAMEs derived from isolated TAG and by dry weight measurements of lyophilized samples collected from 50 mL of media (Fig 1C and 1D). Confounding nutrient limitations caused by high growth rates in mild heat, or unusually high micronutrient requirements, were further controlled in secondary tests by the addition of a double normal quantity of micronutrients and triple the quantity of sodium nitrate. Surprisingly, *C*. *subellipsoidea* was the only algae that retained lipid accumulation during mild heat stress in a high CO_2_, high light, and high nutrient environment (Fig 1B and 1D), with over six-fold greater TAG productions compared to other species tested (Fig 1C). The dry weight doubled over 2 days to 0.34 g per L and only increased to 0.38 by day 5. All other species analyzed grew linearly through 5 days at rates between 0.19 g and 0.28 g dry weight algae per L media per day (Fig 1D). High growth rates leading to algal concentrations in excess of 3 OD_550_ values suggest that other algae identified in the primary screen were possibly nutrient limited by BBM media at 32°C, or that CO_2_ addition ameliorated the phenotype. Further analyses using temperature variation time courses were done using *C*. *subellipsoidea* C169.

### Time course study of biological and biochemical parameters in *C*. *subellipsoidea* C169 varying growth temperature

Triplicate batch cultures with double the normal N content were grown to analyze the effects of heat stress on *C*. *subellipsoidea* using a jacketed bioreactor system. Cultures were grown at room temperature for 5 days and diluted to an OD_550_ of 0.5 prior to starting the temperature condition to control for light intensity, and time courses were run for 5 days with sample taken daily. Growth was monitored and samples measured for TAG, starch, and chlorophyll content. The effects of N starvation were also assessed at 25°C keeping all other testing parameters equal as a reference for the effects of temperature stress.

**Fig 2:**
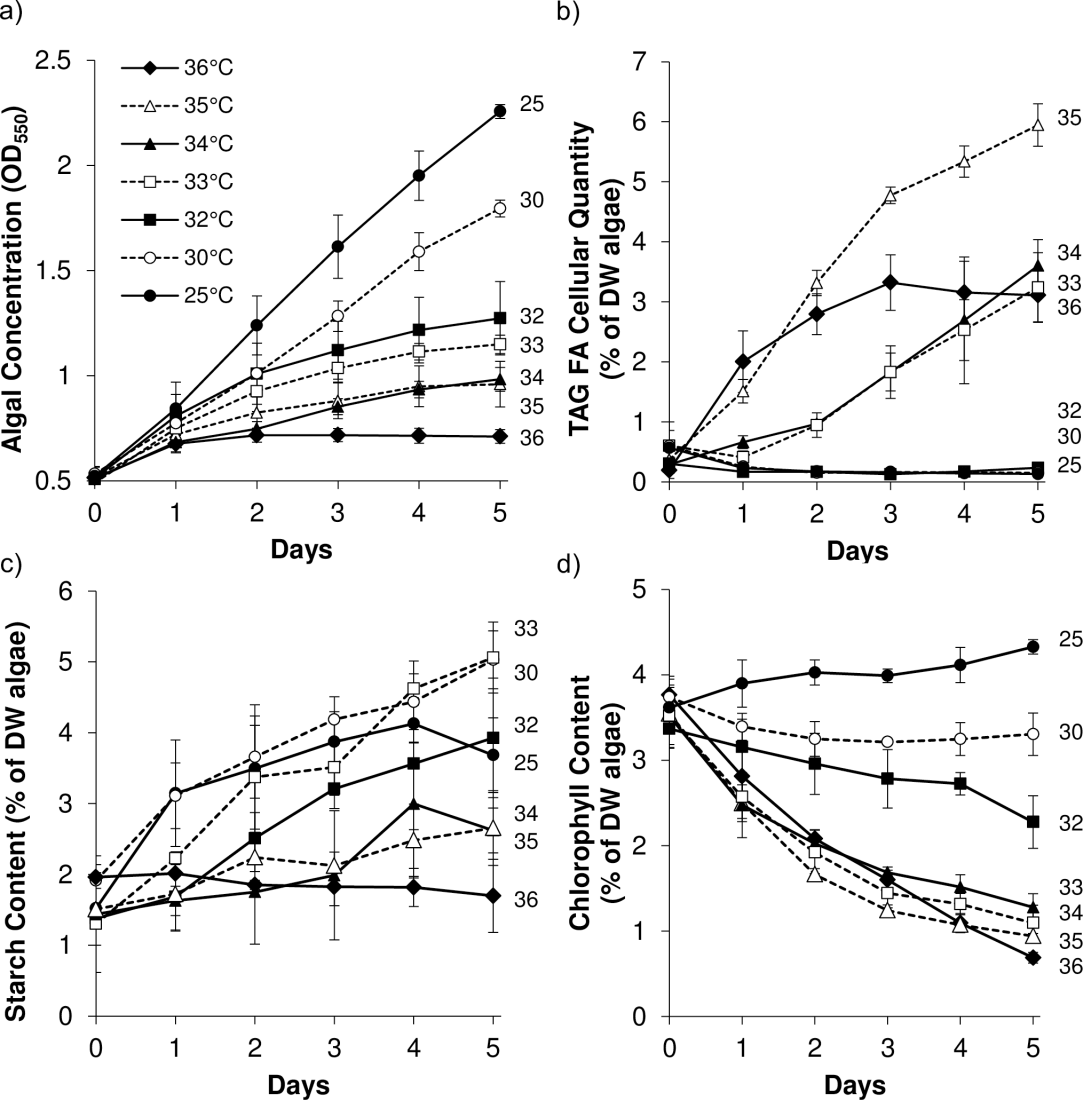
Biochemical characterization of temperature effects on *C*. *subellipsoidea*. Algae was grown in a jacketed bioreactor system with 1% CO_2_ addition varying growth temperature. Experiments were conducted for 5 days from an OD_550_ of 0.5 at the indicated temperature after an initial growth period at 25°C. Growth (a), triglyceride content (TAG; b), starch contet (c), and chlorophyll content (d) were measured. Error bars indicate ± standard deviation (n = 3).

**Fig 3:**
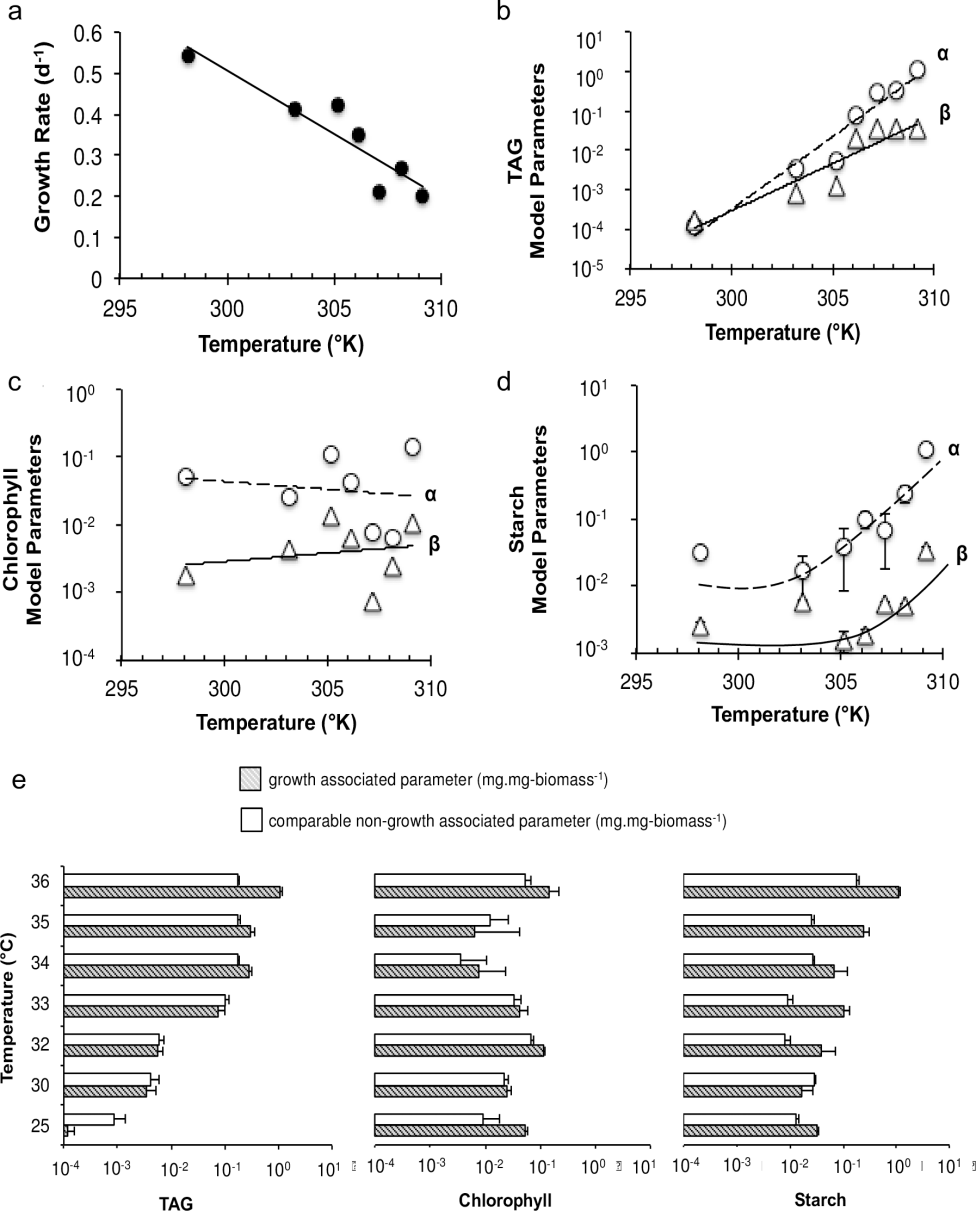
Relationship between model parameters. Change in the kinetic parameters of *C*. *subellipsoidea* C169 with temperature a) specific growth rate, b) TAG, c) chlorophyll, and d) starch (α–growth associated parameter, mg.mg-biomass^-1^; β–non-growth associated parameter, mg.mg-biomass^-1^.d^-1^), e) comparison of growth and converted non-growth kinetic parameters for TAG, chlorophyll and starch production with temperature change.

Most microorganisms’ growth can be modeled using either Monod or Logistic approaches [10, 27, 28]. The Monod model relates growth with limiting substrate utilization. Since autotrophic microalgal growth depends on various limiting substrates (*e*.*g*., carbon dioxide, temperature, light, and carbon source), the Monod model may pose problems in modeling micro-algal growth. A logistic approach, however, allows modeling of growth based on geometrical as well as biological parameters independent of substrate consumption. Therefore, we modeled *C*. *subellipsoidea* growth using a logistic model displaying a lag phase, an initial growth rate, and stationary state with sigmoidal curve [27–29]. To evaluate and compare the maximum specific growth rates of C169 at different temperatures, we performed kinetic modeling using the logistic approach to the experimental growth data using Eq (2). For all temperatures, the growth data fitted well with the model (R^2^ = 0.99; Figs 2A and 3A). The specific growth rates decreased with increase in the temperature (Fig 3E). The Arrhenius temperature relation fitting of the specific growth rate data to Eq (3) indicated that C169 requires ~55.7 kJ.mol^-1^ activation energy to maintain the cellular mechanisms. Culture chlorosis was evident and no further growth occurred after 3 days in 36°C, which was considered the temperature of lethality in the tested conditions.

### Relationship of model-determined kinetic parameters

The Eq (5) fitted well with the experimental data of TAG (Fig 3B), chlorophyll (Fig 3C), and starch (Fig 3D) at all different temperatures (R^2^ = 0.99). Firstly, we converted the non-growth related coefficient (β) values (Fig 3E) to the comparable values of growth related coefficient (α). After 5 days, with the increase in temperature, β values for TAG production were higher than α values, which suggests that the temperature contributes towards a relatively higher non-growth associated TAG production than the growth associated TAG production. The majority of chlorophyll production, however, appeared to be contributed to α rather than β values. Starch shows no significant pattern for α and β with temperature, which proposes that both growth and non-growth related parameters affect starch production.

**Fig 4:**
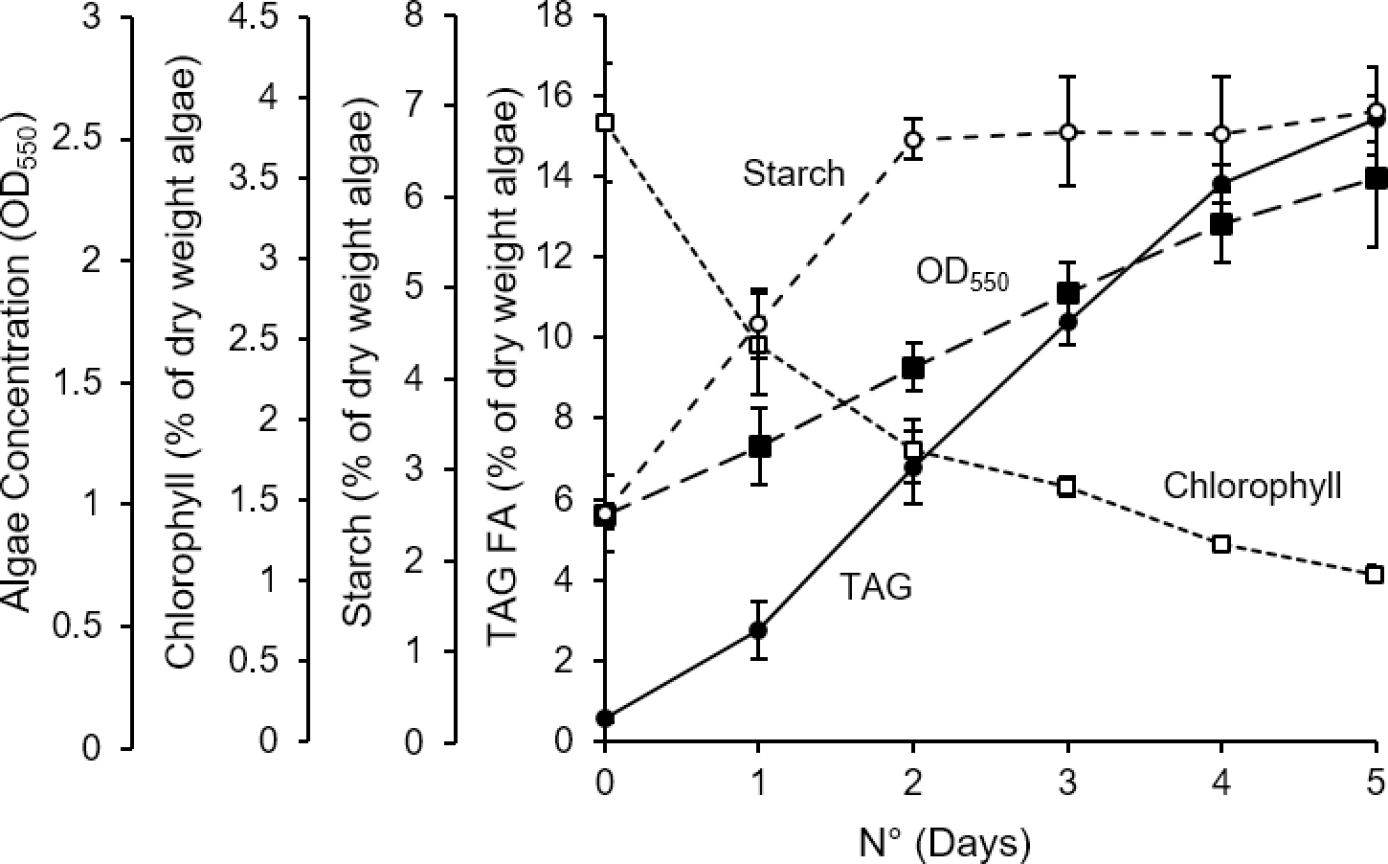
N starvation of *C*. *subellipsoidea* C169. Algae was transferred to an N-free (N°) medium in 25°C to analyze effects on cellular concentration (closed squares), chlorophyll content (open squares), starch content (open circles), and triglyceride accumulation (closed circles) in bioreactor conditions otherwise identical to heat stress testing. Error is ± standard deviations (n = 3).

We observed a positive slope (m = 0.37) for TAG growth coefficient, which indicates an increased TAG levels with temperature. Similarly, starch-related growth coefficient curve has a positive slope (m = 0.21). The starch levels increases at relatively lesser rates than TAG suggesting that the microalgae cellular machinery favored the TAG production than the starch as the temperature rises. On the contrary, a negative slope (m = -0.45) for chlorophyll-growth coefficient suggests a rapid decrease in the levels of chlorophyll in *C*. *subellipsoidea* cells with the temperature. Temperatures associated with TAG synthesis were closely associated with a nearly identical decrease in chlorophyll content (Fig 2D), suggesting commonality. The rate of chlorophyll catabolism was also shared by N starved cultures, so this may be a general factor of the algal stress response leading to TAG accumulation (Fig 4). Unlike lipid accumulation, starch accumulated with increasing temperature starting at a lower temperature (30°C) and reached 5% of dry weight at both 33°C and 35°C. Starch accumulation during N starvation was also much greater than temperature stress, reaching 7% of dry weight.

The association between growth reduction and lipid accumulation is well documented in algae with respect to other abiotic stress conditions [30–32]. In this case, growth was reduced in proportion to increasing temperature and this coincided with increasing quantities of storage TAG, but only after a threshold temperature >32°C (Fig 2A and 2B). The highest accumulation of TAG was up to 6% of dry weight after 5 days at 35°C, 40% of the accumulated TAG under N starvation on a per cell basis (Fig 4), which coincided with the lowest growth rates above the lethal temperature of 36°C (Fig 2A). Our group has previously demonstrated a correlation between the degree of N limitation, growth retardation, and lipid accumulation in *C*. *subellipsoidea* grown in a chemostatic continuous culture system [10]. Compared to optimal growth at 25°C there were growth reductions of 27% and 56% in 30°C and 32°C, respectively (Fig 2A) which did not increase TAG accumulation. In comparison, Wang *et al*. [33] showed that lipid content is linear with respect to growth reduction caused by N limitation for *C*. *subellipsoidea*. In that study, a 33% reduction in N availability from the normal 3mM in BBM media to 1mM reduced growth by 33% and increased lipid content by 5.3 fold. Reducing N by 83% reduced growth by 53% and increased lipid content by 6.6 fold. The implication of this and our own published chemostat data [10] that growth and lipid content are directly linked is clearly an artifact of using N limitation as a means of reducing growth when viewed in the context of the heat stress results. TAG accumulation can be very specifically induced using heat stress with this species and factors such as those related to the reduction in growth rate isolated from the regulatory and metabolic state causing that accumulation, e.g. by analyzing cells grown at 32°C compared to 33°C.

### Analysis of incremental temperature increases

The pattern of TAG accumulation in *C*. *subellipsoidea* is similar to the pattern of expression of *Chlamydomonas reinhardtii* small heat shock proteins HSP22A, HSP22B, HSP22E, and HSP22F, which are upregulated at a specific temperature between 32°C and 36°C [34]. Kobayashi *et al*. [34] also characterized two HSP22 protein family homologues in a heat tolerant alga, *Cyanidioschyzon merolae*. This species has an optimum growth temperature of 35°C, about 10°C higher than both *C*. *subellipsoidea* and *C*. *reinhardtii*. Interestingly, the small heat shock proteins from *C*. *merolae* are upregulated in a 10°C higher temperature range between 42°C and 46°C, suggesting that an algal heat stress response occurs at a specific threshold temperature related to the optimal growth temperature of the species rather than a jump in temperature as is the case with higher plants [35, 36].

**Fig 5:**
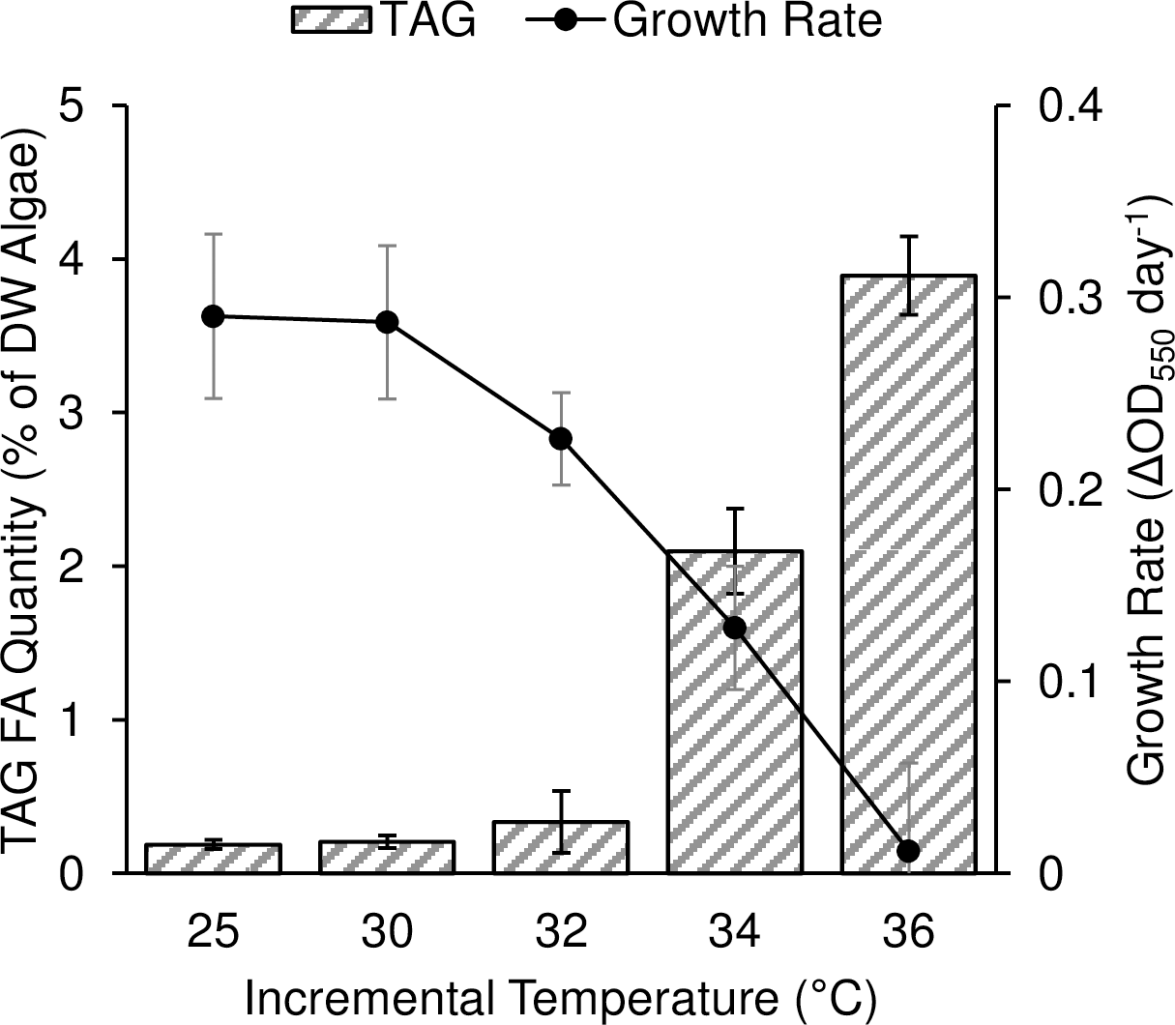
Oil content and growth rate for *C*. *subellipsoidea* C169 during incrementally increasing growth temperatures. Algae was grown in a jacketed bioreactor with 1% CO_2_ for 1 day at each successively increasing temperature (25°C, 30°C, 32°C, 34°C, and 36°C). Triglyceride associated fatty acid (TAG FA) was quantified by GC-MS (striped bars) and growth rates derived from the change in cell density measured as OD (ΔOD_550_ per day). Error bars are ± standard deviation (n = 4).

In order to test this hypothesis in *C*. *subellipsoidea*, replicate cultures were grown in succession in the same conditions as the modeling data starting at 25°C and an OD_550_ of 0.5, but with 1-day duration incremental temperature increases rather than constant temperatures. An initial jump of 5°C from 25°C to 30°C was followed by 2°C increases to 36°C (Fig 5A). Following 1 day intervals at the prescribed temperatures, growth rates were determined by OD measurements and TAG accumulation by GC-MS (Fig 5B and 5C). Growth reduction was coincident to TAG accumulation, beginning with a very slight increase in TAG and minor growth retardation at 32°C, and increasing in severity through 36°C. This data demonstrates that the TAG accumulation in *C*. *subellipsoidea* in response to heat stress is induced at a specific temperature slightly higher than 32°C, and is likely related to heat shock responsive elements known in other algae.

Yang *et al*. [18] speculated that the mild heat stress induced oleaginous phenotype in *E*. *oleoabundans* was related to an adaptation to extreme temperature shifts encountered in its indigenous desert habitat. *C*. *subellipsoidea* was originally isolated in the Antarctic, which shares the shift to much lower temperatures at night. Unlike continuous heat-adapted (e.g. hot springs) or cold-adapted (e.g. snow) algae, the heat-stress oleaginous phenotype may somehow be related to environmental temperature variability, and thus may not be universal among green microalgae.

### Free amino acid and protein synthesis during heat stress

The percentage ^15^N labeling of free amino acids was measured after a 1-day addition of ^15^N-labeled ammonia to bioreactor-grown cultures, either switching to 35°C or maintaining the temperature at 25°C (Table 2). The total protein pool was also extracted and hydrolyzed. Labeled amino acid incorporation into proteins was measured to determine if individual ribosomes may be differentially affected by heat, and as a proxy for overall protein synthesis rates (Table 2). A reduction in the synthesis of any amino acids could reduce protein synthesis and algal growth, thus replicating the N starvation phenotype. There were no statistically significant changes in ^15^N labeling of any free amino acid measured resulting from heat stress (ANOVA; p<0.05). Free amino acids were between 40% and 80% ^15^N-labeled with an average of 57% (Table 2). Tyrosine had the lowest and glutamine the highest percentage labeling, with proline the next highest. The high temperature environment retarded growth by half. Cultures were normalized to an OD_550_ of 0.51 ± 0.04 for both conditions. The control batches reached an OD_550_ of 1.0 ± 0.08 and the high temperature batches 0.74 ± 0.08, and the average labeling of protein-bound amino acids was 38% ± 1% and 27% ± 3%, respectively (Fig 5c). The empirical ^15^N-percent labeling was much higher at 35°C than the theoretical labeling of 19% based on the change in OD_550_ values, suggesting that protein turnover is significantly increased under heat stress. Heat stress by these measurements did not adversely affect N assimilation, the synthesis of individual amino acids, the incorporation of specific amino acids into proteins, or the cellular rate of protein synthesis.

**Table 2:** Amino acid and protein synthesis during heat stress.

	Free AA		Protein-Bound AA	
	(% labeled)		(% labeled)	
**Amino Acid (AA)**	**25⁰C**	**35⁰C**	**25⁰C**	**35⁰C**
**Alanine**	57 ± 12	67 ± 8	40 ± 2	31 ± 1
**Asparagine**	43 ± 10	50 ± 4	36 ± 1	32 ± 1
**Aspartate**	57 ± 13	68 ± 8	N/D	N/D
**Glutamine**	72 ± 10	80 ± 7	N/D	N/D
**Glutamate**	57 ± 12	67 ± 8	N/D	N/D
**Glycine**	50 ± 12	57 ± 8	37 ± 1	25 ± 1
**Isoleucine**	53 ± 12	64 ± 8	38 ± 1	27 ± 1
**Leucine**	45 ± 12	57 ± 14	37 ± 1	26 ± 1
**Methionine**	50 ± 9	56 ± 5	38 ± 1	24 ± 1
**Phenylalanine**	46 ± 4	54 ± 2	N/D	N/D
**Proline**	58 ± 13	69 ± 9	40 ± 1	28 ± 1
**Serine**	53 ± 12	62 ± 7	38 ± 1	25 ± 3
**Threonine**	53 ± 9	60 ± 5	38 ± 1	26 ± 1
**Tyrosine**	42 ± 7	48 ± 4	N/D	N/D
**Valine**	51 ± 10	60 ± 8	37± 1	26 ± 1
**Average**	52 ± 4	62 ± 4	38± 1	27 ± 3

% labeled, percentage ^15^N labeling measured by GC-MS after 1 day at given temperature; N/D, not detected.

### Effects of mild temperature stress and N starvation on the production and allocation of fatty acids to the TG pool

Fatty acids were quantified by mass spectroscopy as methyl ester derivatives of isolated TG lipids and total lipid extracts. The effect of a range of temperatures on the flow of C through specific fatty acids was analyzed using this data relative to N starvation. The quantity of molecules of each fatty acid per liter of media was used to assess C flow in terms of culture volume per unit time. The resulting flow diagram effectively demonstrates key results of lipid profiling but cannot be considered flux diagrams as they do not account for any degradation by fatty acid oxidation, conversion by elongation or acyl-chain shortening, excretion, or incorporation into waxes or structural glycolipids. They do reflect the applicable measurements for industrial applications, namely the empirical change in the quantities of fatty acids over time under the conditions tested.

**Fig 6:**
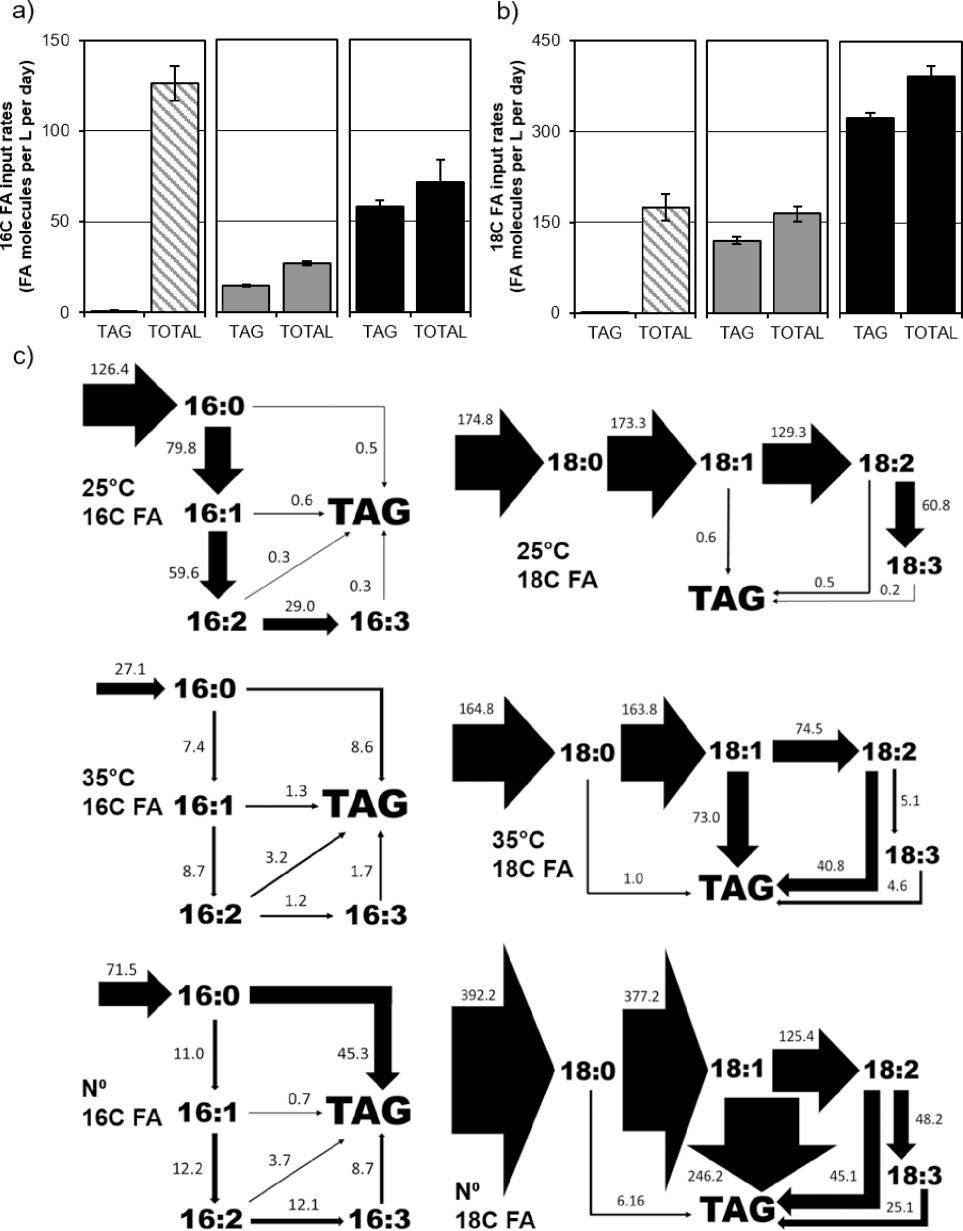
Rates of fatty acid synthesis and input into triglycerides during heat and N stress. Fatty acids from total lipid extracts and silica column isolated triglycerides (TAGs) were quantified by GC-MS over 5-day time courses in normal conditions (25°C), heat stress (35°C), and N starvation (N°) and the data converted to average rates (10^7^ molecules per liter of growth medium per day). Graphs above show the total rates of FA synthesis compared to the rates of FA incorporation into TAG for 16C FA (a) and 18C FA (b) pools at 25°C (diagonally striped bars), 35°C (gray bars), and N° (black bars). Figure below depicts averaged influx and efflux rates (indicated bedside arrows) for specific FA’s derived from quantitative data with arrow widths directly proportional to the corresponding rate.

Several effects specific to heat stress and in common between heat and N stress were revealed by this analysis. The total input rates of all 16C or 18C fatty acids compared to the flux into TAG pools alone show that N starvation (N°) results in a 145% increase in total FA synthesis due to a 224% increase in 18C FA and a decrease of 43% in 16C FA production (Fig 6A and 6B). In contrast, growth in 35°C does not induce FA synthesis. Instead it reduces C flux into 16C FA by 79% and 18C flux remains equal to the 25°C control. The exaggerated input into 18C FA in the N° state mainly flows into oleic acid (18:1) which is completely removed by desaturation to linolenic acid (18:2) or sequestration into TAG (Fig 6C). Possibly in reaction to the effects of heat on the viscosity of membrane lipids, in 35°C the conversion from 18:2 to α-linoleic acid (18:3) is greatly reduced. This rate is 8% compared to 25°C and 11% compared to N° (Fig 6C). Movement of 18:2 into TAG is relatively equal between 35°C and N° cultures at 40.8 and 45.1 (10^7^ molecules per liter of growth medium per day).

During heat stress, there is a small net influx of 18:1 into membrane lipids resulting in an enrichment of monounsaturated FA, which could have the effect of decreasing membrane fluidity (Fig 6C). This may be the result of the reduced or restricted conversion or sequestration of 18:1, which is known to allosterically inhibit FA synthesis in higher plants [37]. In N°, there is no net influx into 18:1, and it may be that the 18:1 pool produced during heat stress results in the FA synthesis rates being lower compared to N°. In this scenario, both heat and N stress result in the reduction of 16C FA production, and whereas in N stress there is an overall induction in FA synthesis because of the increased production of 18C FAs, in heat stress the inhibition of the conversion of 18:2 to 18:3 ultimately results in a buildup of 18:1, which in turn allosterically inhibits FA synthesis. Further research into the regulation of FA synthesis in the context of heat stress is needed to explore this hypothesis. Regardless of the cause, the data are clear in that TAG synthesis during heat stress derives from a redistribution of flux away from membrane or other lipids rather than an overall increase in the FA synthetic rate, as is the case with N stress.

## Conclusion

One of a total of 42 algae screened, *Coccomyxa subellipsoidea* C169, demonstrated a 6-fold greater heat induced TAG production phenotype in a high CO_2_ bioreactor system. Growth, chlorophyll content, TG and starch accumulation were analyzed and the results mathematically modeled. Although growth was greatly reduced between 25°C and 32°C, lipid only accumulated in a narrow range between 32°C and 36°C. This is unlike N stress where growth and lipid droplet production are negatively proportional. Also distinct from N starvation, analyses using ^15^N demonstrated that amino acid and protein synthesis were not greatly affected by 35°C. Heat stress results in LD accumulation mainly by redirecting 18C FAs away from anabolic membrane lipid pathways and into TG synthesis, and is distinct from N deprivation which results in an overall increase in fatty acid synthesis. The data indicates that heat stress is distinct form N stress in the mechanism of TAG production. Future studies could provide unique insights on the metabolic regulation underpinning stress induced lipid droplet production in algae.

## Supporting information

S1 File Rate analysis of GC-MS lipid profile quantitative data. Fatty acids quantified by GC-MS over 5-day time courses in normal conditions (25°C), heat stress (35°C), and N starvation (N°) converted to average rates (10^7^ molecules per liter of growth medium per day) using linear regression analyses.
(XLSX)

S1 Table Model parameters for lipid, chlorophyll, and starch after fitting experimental data with respective model equations. Software used was Mathematica ver. 11.0.1.0.
(DOCX)
