# Supplementary material for: Induction of oil accumulation by heat stress is metabolically distinct from N stress in the green microalgae Coccomyxa subellipsoidea C169
Source: PLoS One. 2018 Sep 27;13(9):e0204505. doi: 10.1371/journal.pone.0204505 (PMC6160078; doi:10.1371/journal.pone.0204505)
Supplement: S1 Table — Software used was Mathematica ver. 11.0.1.0. (DOCX) [file pone.0204505.s002.docx]

**Table 1.** Model parameters for lipid, chlorophyll, and starch after fitting experimental data with respective model equations (software–Mathematica ver. 11.0.1.0 ).

| **Temp** (°C) | $\boldsymbol{\mu}_{\boldsymbol{max}}$ (day^-1^) | **Lipid (TAG)** | | **Chlorophyll** | | **Starch** | |
| --- | --- | --- | --- | --- | --- | --- | --- |
|  |  | $\boldsymbol{\alpha}$, 10^-4^  (mg/mg-biomass) | $\boldsymbol{\beta}$**,** 10^-4^  (mg/(mg-biomass.d)) | $\boldsymbol{\alpha}$**,** 10^-4^  (mg/mg-biomass) | $\boldsymbol{\beta,}$ 10^-4^  (mg/(mg-biomass.d)) | $\boldsymbol{\alpha}$**,** 10^-4^  (mg/mg-biomass) | $\boldsymbol{\beta,}$ 10^-4^  (mg/(mg-biomass.d)) |
| 25 | 0.54 | 3.96 ± 1.18 | 1.75 ± 0.50 | 522 ± 67 | 18.6 ± 7.7 | 326 ± 18 | 25.1 ± 4.7 |
| 30 | 0.42 | 33.8 ± 18.3 | 8.35 ± 3.73 | 252 ± 49 | 44.2 ± 9.9 | 167 ± 10.8 | 58.1 ± 1.9 |
| 32 | 0.41 | 54.5 ± 15.7 | 12.1 ± 2.7 | 11.3 ± 1.1 | 13.6 ± 1.9 | 397 ± 32 | 15.4 ± 0.56 |
| 33 | 0.35 | 739 ± 270 | 200 ± 39 | 413 ± 181 | 64.9 ± 26.1 | 1,020 ± 230 | 18.2 ± 4.9 |
| 34 | 0.27 | 2,870 ± 360 | 345 ± 30 | 74.9 ± 16.4 | 7.2 ± 3.9 | 686 ± 59 | 53.6 ± 4.3 |
| 35 | 0.21 | 3,090 ± 610 | 339 ± 48 | 63.6 ± 6.3 | 24.8 ± 8.8 | 2,430 ± 690 | 51 ± 5.5 |
| 36 | 0.20 | 10,900 ± 800 | 344 ± 35 | 14.3 ± 7.4 | 10.7 ± 3.1 | 1,080 ± 120 | 348 ± 49 |
